# In Silico and In Vitro Potential Antifungal Insights of Insect-Derived Peptides in the Management of *Candida* sp. Infections

**DOI:** 10.3390/ijms26157449

**Published:** 2025-08-01

**Authors:** Catarina Sousa, Alaka Sahoo, Shasank Sekhar Swain, Payal Gupta, Francisco Silva, Andreia S. Azevedo, Célia Fortuna Rodrigues

**Affiliations:** 1 Associate Laboratory i4HB—Institute for Health and Bioeconomy, University Institute of Health Sciences—CESPU, 4585-116 Gandra, Portugal; a29672@alunos.cespu.pt; 2 Department of Skin & VD, Institute of Medical Sciences & SUM Hospital, Siksha ‘O’ Anusandhan Deemed to be University, Bhubaneswar 751003, Odisha, India; alakasahoo81@gmail.com; 3 Research and Development Division, Salixiras Research Private Limited, Bhubaneswar 751012, Odisha, India; 4 Division of Microbiology & NCDs, ICMR-Regional Medical Research Centre, Bhubaneswar 751023, Odisha, India; 5 Department of Biotechnology, Graphic Era Deemed to be University, Dehradun 248001, Uttarakhand, India; payal.hillview@gmail.com; 6 Department of Pharmaceutical Sciences, University Institute of Health Sciences (IUCS-CESPU), 4585-116 Gandra, Portugal; francisco.silva@iucs.cespu.pt; 7 UCIBIO—Applied Molecular Biosciences Unit, Translational Research Laboratory, University Institute of Health Sciences (1H-TOXRUN, IUCS-CESPU), 4585-116 Gandra, Portugal; asazevedo@fe.up.pt; 8 LEPABE—Laboratory for Process Engineering, Environment, Biotechnology and Energy, Faculty of Engineering, University of Porto, Rua Dr. Roberto Frias, 4200-465 Porto, Portugal; 9 ALiCE—Associate Laboratory in Chemical Engineering, Faculty of Engineering, University of Porto, Rua Dr. Roberto Frias, 4200-465 Porto, Portugal

**Keywords:** *Candida* spp., *Candida albicans*, *Candida tropicalis*, *Candida glabrata*, *Candida parapsilosis*, insect peptides, *Candida* infections, antifungal resistance, antifungal activity, in silico investigations, phylogenetic tree prediction

## Abstract

The worldwide increase in antifungal resistance, particularly in *Candida* sp., requires the exploration of novel therapeutic agents. Natural compounds have been a rich source of antimicrobial molecules, where peptides constitute the class of the most bioactive components. Therefore, this study looks into the target-specific binding efficacy of insect-derived antifungal peptides (*n* = 37) as possible alternatives to traditional antifungal treatments. Using computational methods, namely the HPEPDOCK and HDOCK platforms, molecular docking was performed to evaluate the interactions between selected key fungal targets, lanosterol 14-demethylase, or LDM (PDB ID: 5V5Z), secreted aspartic proteinase-5, or Sap-5 (PDB ID: 2QZX), N-myristoyl transferase, or NMT (PDB ID: 1NMT), and dihydrofolate reductase, or DHFR, of *C. albicans*. The three-dimensional peptide structure was modelled through the PEP-FOLD 3.5 tool. Further, we predicted the physicochemical properties of these peptides through the ProtParam and PEPTIDE 2.0 tools to assess their drug-likeness and potential for therapeutic applications. In silico results show that Blap-6 from *Blaps rhynchopeter* and Gomesin from *Acanthoscurria gomesiana* have the most antifungal potential against all four targeted proteins in *Candida* sp. Additionally, a molecular dynamics simulation study of LDM-Blap-6 was carried out at 100 nanoseconds. The overall predictions showed that both have strong binding abilities and are good candidates for drug development. In in vitro studies, Gomesin achieved complete biofilm eradication in three out of four *Candida* species, while Blap-6 showed moderate but consistent reduction across all species. *C. tropicalis* demonstrated relative resistance to complete eradication by both peptides. The present study provides evidence to support the antifungal activity of certain insect peptides, with potential to be used as alternative drugs or as a template for a new synthetic or modified peptide in pursuit of effective therapies against *Candida* spp.

## 1. Introduction

*Candida* spp. infections are rising all over the world and present a significant challenge to global health due to its prevalence as a major opportunistic fungal pathogen responsible for causing infections stretching from superficial mucosal infections to life-threatening systemic infections, especially in immunocompromised individuals [1,2,3,4,5]. *C. albicans* infections are prevalent in hospital settings and are a leading cause of invasive candidiasis, which has high morbidity and mortality rates [1,2,3,4,5]. However, there is an increase in more incident non-*Candida albicans Candida* species (NCACs) infections, such as *Candida tropicalis, Candida glabrata*, and *Candida parapsilosis* [6]. As highlighted by the World Health Organization’s inaugural fungal priority pathogens list, *Candida auris* and *C. albicans* are among the four most critical pathogens, and *C. tropicalis*, *C. glabrata* (*Nakaseomyces glabrata*), and *Candida parapsilosis* were listed in the high-priority group, underscoring the urgent need for new treatment options [7]. The emergence of antifungal resistance, such as resistance to azoles and echinocandins, has further complicated treatment options and highlighted the urgent need for new therapeutic strategies [8,9,10]. This pathogen’s adaptability, biofilm-forming capability, and virulence factors make it a persistent threat, emphasizing its impact on global healthcare systems and the need for innovative research in antifungal drug development [3,11,12]. Therefore, exploring new antifungal agents against *C. albicans* and NCACs is essential due to rising drug resistance and limited treatment options [9,10].

As an alternative therapeutic option, natural-sources-derived metabolites and peptides offer a promising alternative against *C. albicans*, especially in the face of increasing resistance to standard antifungal drugs [11,13,14,15,16]. Particularly, bioactive peptides, derived from various natural sources including insects, plants, and marine organisms, exhibit potent antifungal properties with unique mechanisms of action, such as disrupting fungal cell membrane permeabilization and subsequent cell death and modulating immune responses [17,18,19,20,21,22,23]. Insect-derived host defense peptides and lipopeptides have garnered attention for their potential as antifungal agents [24]. The antimicrobial properties of insect-derived peptides have been studied, with research demonstrating their efficacy against a broad spectrum of fungal pathogens, including *Candida* species [24]. *Candida* infections have been a persistent challenge, with the emergence of drug-resistant strains and the difficulty of treating biofilm-associated infections. The potential of designed antimicrobial peptides as a novel approach to addressing the challenges posed by *Candida* infections can be a path to the discovery of novel and effective antifungal drugs. These laboratory-synthesized peptides, designed using naturally occurring antimicrobial peptides, have shown promising results in terms of direct antifungal activity and the disruption of fungal biofilms.

These molecules, composed of cationic peptides conjugated to aliphatic acids, exhibit a high degree of antifungal bioactivity. These peptides, including cecropins, defensins, attacins, and others, are known for their diverse mechanisms of action, such as disrupting fungal cell membranes, inhibiting biofilm formation, and modulating immune responses [18,19,25]. This unique mode of action offers a potential advantage over traditional antifungal drugs, as it may be less prone to the development of resistance. Moreover, due to their natural origin and selective toxicity, antifungal peptides present a lower risk of adverse effects, positioning them as viable candidates for developing safer, more effective treatments against *C. albicans* and NCACs infections [18,25,26]. These peptides can effectively disrupt *C. albicans* cell walls, inhibit biofilm formation, and offer immunomodulatory benefits, making them highly effective against resistant strains [18,25,26,27].

In summary, insect-derived peptides are advantageous for their high specificity, minimal cytotoxicity, and effectiveness against resistant strains, making them promising next-generation antifungal agents, addressing an urgent need for effective treatments [18,25,26,27]. As a result, insect-derived host defense peptides have garnered attention and offer a potential advantage over traditional antifungal drugs, as they may be less prone to the development of resistance. In fact, insect peptides often have broad-spectrum bioactivity and different therapeutic effects against different pathogens. It is more important to study their target-specific efficacy. Similar to small therapeutic molecule-based drug discovery, the process of designing or isolating bioactive peptides and experimentally validating them individually against a panel of pathogens can be more challenging and resource-intensive. In this perspective, as a first approach, computational methods help speed up the drug discovery process by enabling quick screening and optimization of potential candidates [28,29,30]. This approach is not only cost-effective and time-efficient, allowing for high-throughput screening of peptide candidates, but it also facilitates the identification of promising molecules before synthesis and experimental validation [28,29,30]. The initial screening is helpful in improving peptide sequences that enhance antifungal activity while also predicting possible resistance mechanisms in *Candida* spp. Ultimately, this exploration accelerates drug discovery, paving the way for targeted therapies that effectively combat fungal infections while minimizing off-target effects and addressing the pressing issue of antifungal resistance [28,29,30]. Ongoing research in this field aims to further elucidate the molecular mechanisms underlying the differential cell-specificity of these host defense molecules as well as to optimize their efficacy and safety for potential clinical applications [31,32]. Following the same steps, this study uses molecular docking and molecular dynamics (MD) simulation to find the most potent insect peptides against *Candida* spp. and to look into their physical and chemical properties. Following the computational studies, we selected two of the thirty-seven analyzed peptides with best in silico profiles—Blap-6 and Gomesin—and conducted in vitro assays on *C. albicans*, *C. tropicalis*, *C. glabrata*, and *C. parapsilosis* to validate the predicted results and assess the efficacy of these promising antifungal molecules.

## 2. Results

### 2.1. In Silico Analysis and In Vitro Tests

#### 2.1.1. Antifungal Insect Peptide Library Preparation from Existing Reports

Based on our hypothesis and an extensive literature search, thirty-seven insect-derived peptides with antifungal activity were identified that belong to 29 different insects, including *Apis mellifera*, *Bombyx mori*, *Drosophila melanogaster*, *Oxysternon conspicillatum*, *Stomoxys calcitrans*, *Tenebrio molitor*, and other insects. Peptides were investigated for their antifungal activity against 38 pathogenic fungal strains such as *C. albicans*, *C. glabrata*, *C. tropicalis*, *Aspergillus fumigatus*, *A. parasiticus*, or *Cryptococcus neoformans*, and the minimum fungicidal concentration (MFC) values were within 0.1 to >200 µM (Table S1). According to MFC values, Blap-6, cecropins A & C, drosomycin, gomesin, metchnikowin, and stomoxyn had the best antifungal potential against the tested fungal strains, with MFC values < 1 µM. The recorded peptide sequence ranged from 8 to 45 amino acids, with diapausin-1 being the longest at 45 amino acids and jelleine-I being the smallest at 8 amino acids (Table S1).

#### 2.1.2. Three-Dimensional Peptide Structure Modelling and Molecular Docking Study

**Figure 1:**
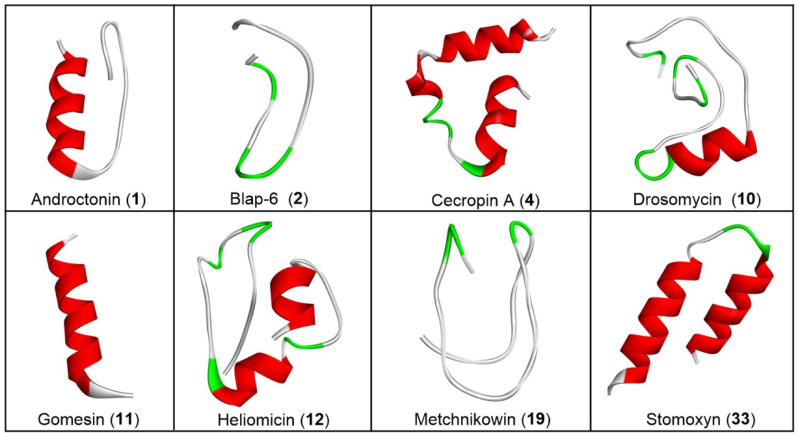
Three-dimensional (3D) structure of selected bioactive insect peptides. Red represents spiral shaped α-helices; green represents arrow-like shaped β-strands; and white/grey represents unstructured flexible regions that not adopt helix or strand conformation. The software BIOVIA Discovery Studio Visualizer-2019 (academically free version) was used for this 3D presentation.

**Figure 2:**
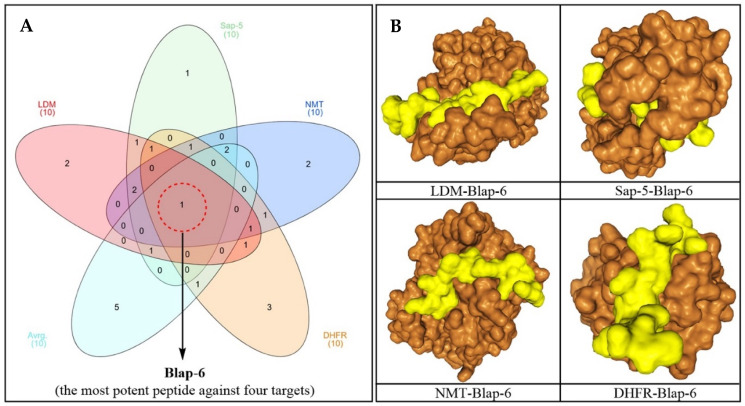
(**A**) Venn diagram of the top ten most potential peptides based on individual and average docking scores, and Blap-6 was selected as the most potential and common peptide against all targets (LDM, Sap-5, NMT and DHFR, *C. albicans* enzymes). (**B**) Molecular interaction of Blap-6 against four targets with the most potential docking poses (software for 3D presentation: BIOVIA Discovery Studio Visualizer-2019). Blap-6 is represented in yellow and the targets in orange.

In accordance with the requirements, a 3D structure for each peptide was generated based on its sequence for the docking study (Figure 1). The recorded molecular docking score of each peptide was compiled, as shown in Table S1. Based on both individual and average docking scores, Blap-6 emerged as the most promising peptide, with docking scores (kcal/mol) of −299.86 against LDM, −290.09 against Sap-5, −245.30 against NMT, and −222.95 against DHFR. The average docking score for each target was −264.53, as shown in Table S1. We primarily selected the top ten potential antifungal peptides based on their docking scores and then used a Venn diagram (Figure 2A) to identify the common peptide. The Venn diagram clearly indicated that Blap-6 (2) was the most potential candidate against all four targets, and the 3D molecular interaction of Blap-6 with all four targets is presented in Figure 2B. Noted experimentally validated antifungal activity was recorded against all the fungal strains. Molecular docking studies helped in selecting the best one from a series and in guiding further experimental studies with limited candidates with a higher rate of translational success. However, it is important to note that this is a theoretical approach, based on binding energy, and further experiments are mandatory [29,33]. Consequently, while in silico studies guided the process with limited resources, their results did not recommend consumption or human use [29,33].

#### 2.1.3. Molecular Dynamics Simulation Study

**Figure 3:**
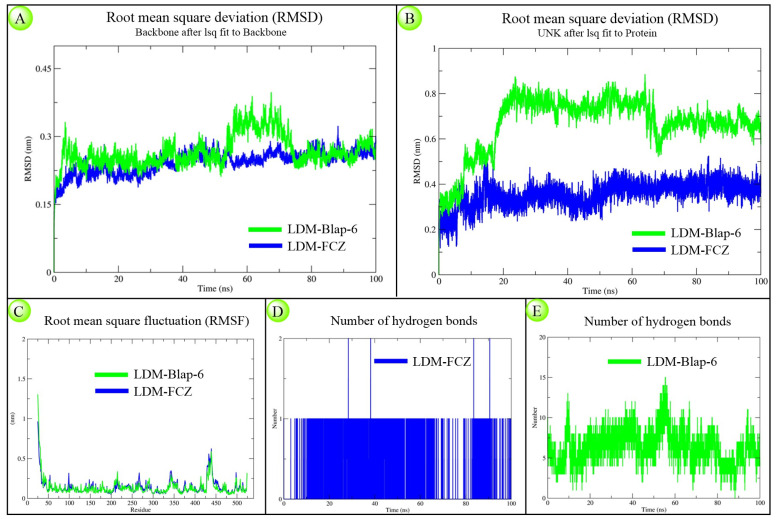
Molecular dynamics (MD) simulation result analysis of LDM-Blap-6 and LDM-FCZ docking complexes over a 100 ns timescale towards observing the structural stability and binding dynamics. (**A**) Backbone RMSD plot showing the overall protein structural deviation; (**B**) ligand RMSD plot indicating the stability of the ligand within the binding pocket; (**C**) RMSF plot representing residue-wise flexibility of the protein; (**D**) number of hydrogen bond interactions in the LDM-FCZ system; and (**E**) number of hydrogen bond interactions in the LDM-Blap-6 system.

The structural and kinetic stability of LDM-Blap-6 and LDM-FCZ complexes over 100 ns was observed according to generated RMSD (both backbone protein and ligand), RMSF, and number of hydrogen bonds plots. The backbone RMSD plots showed that the peptide complex displayed higher fluctuations between 0–8 ns and 53–73 ns, but both complexes stabilized similarly by the end of the simulation (Figure 3A). The ligand RMSD plot ranged from 0.2–0.9 nm for Blap-6 and 0.1–0.5 nm for the FCZ system (Figure 3B). RMSF plots indicated comparable residue-wise fluctuations in both systems (Figure 3C). Hydrogen bond analysis revealed consistent interaction for FCZ with a single H-bond throughout (Figure 3D), while Blap-6 formed 2–15 H-bonds dynamically with LDM (Figure 3E). Due to their distinct chemical nature, both ligands exhibited different deviation patterns. Overall, the findings suggest that despite structural differences, Blap-6 demonstrated comparable stability to FCZ under simulated physiological conditions.

#### 2.1.4. Physicochemical Property Analysis of Peptides

**Figure 4:**
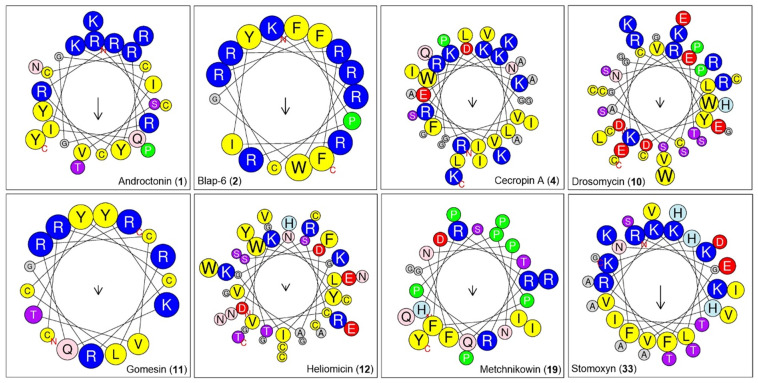
The helical wheel representations of selected bioactive insect-derived peptides illustrate their physicochemical properties. Color coding is based on the tool’s indication: blue represents hydrophobic residues, red indicates residues with net zero charge, pink denotes polar residues, and yellow corresponds to non-polar residues.

Further, we predicted the physicochemical profiles of the peptides as per their sequence (Table 1). We found that jelleine_I (954.18 Da) was the low-molecular-weight insect peptide, while oxysterlin_4 (7438.83 Da) was the highest-molecular-weight peptide from the list. The molecular weight of the most potential peptide, Blap-6, was 2400.89 Da. The pI value of the peptides ranged from 4 to 13, while the predicted pI value of Blap-6 was 12.13. Based on NC (total negatively charged residues), oxysterlin 2 had a higher number (8), while oxysterlin 4 had a higher PC (total positively charged residues) compared to the predicted records (Figure 4; Table 2). However, the instability index (II) revealed that Blap-6 exhibited a more unstable profile when compared with the others. The GRAVY (hydrophobicity/hydrophilic) profiles revealed that coprisin, jelleine I and II, lasioglossin III, MAF-1A, melectin, NDBP-5.7, papiliocin, polybia-MPI, polybia-MPII, protonectin, tenecin 1, and ToAP2 were hydrophobic, while the remaining peptides, as indicated by the positive value, were hydrophilic in nature.

This is a promising sign for Blap-6. The predicted half-life (h) profiles showed that most peptides have a half-life period of more than one hour. Overall, some of the peptides demonstrated drug-favorable profiles with higher translational ability. However, prolonged exposure or delayed excretion can lead to adverse effects. Overall, some of the peptides demonstrated drug-favorable profiles with higher translational ability (Table 1 and Table 2). Alternatively, similar to small drug candidates, we could implement peptide modification or chemical hybridization to enhance the appropriate physicochemical profiles [34,35,36]. We know that, in preliminary investigation, several peptides show therapeutic values, but in later stages, like in vivo models or clinical trial stages, most of them fail to translate as mainstream drugs due to having inadequate drug ability profiles, such as poor stability, low bioavailability, lack of targeting ability, and toxicity profiles. Therefore, to overcome these limitations, there are many innovative and ingenious approaches, like structural modification or chemical conjugation, to turn them into ideal drug candidates. Peptides can be modified through some mechanisms such as peptide backbone modification via cyclization, D-amino acid substitution, and N-methylation; inserting a peptide side chain via lipidation and PEGylation; chemical conjugation via drug–peptide conjugation and nanocarrier attachment; non-natural amino acid incorporation via beta-amino acid and fluorinated amino acids; prodrug design via enzyme-activated prodrugs and self-assembly peptides; and utilizing recombinant protein production to improve the peptide stability and penetration ability, reduce its toxicity, and, overall, improve its therapeutic index for higher translational success and to enhance its pharmacokinetic, pharmacodynamic, and physicochemical characteristics, which will impact the success of peptides as new potential drugs against *Candida* sp.

#### 2.1.5. Homologues-Cum-Phylogenetic Tree Analysis of Peptides

**Figure 5:**
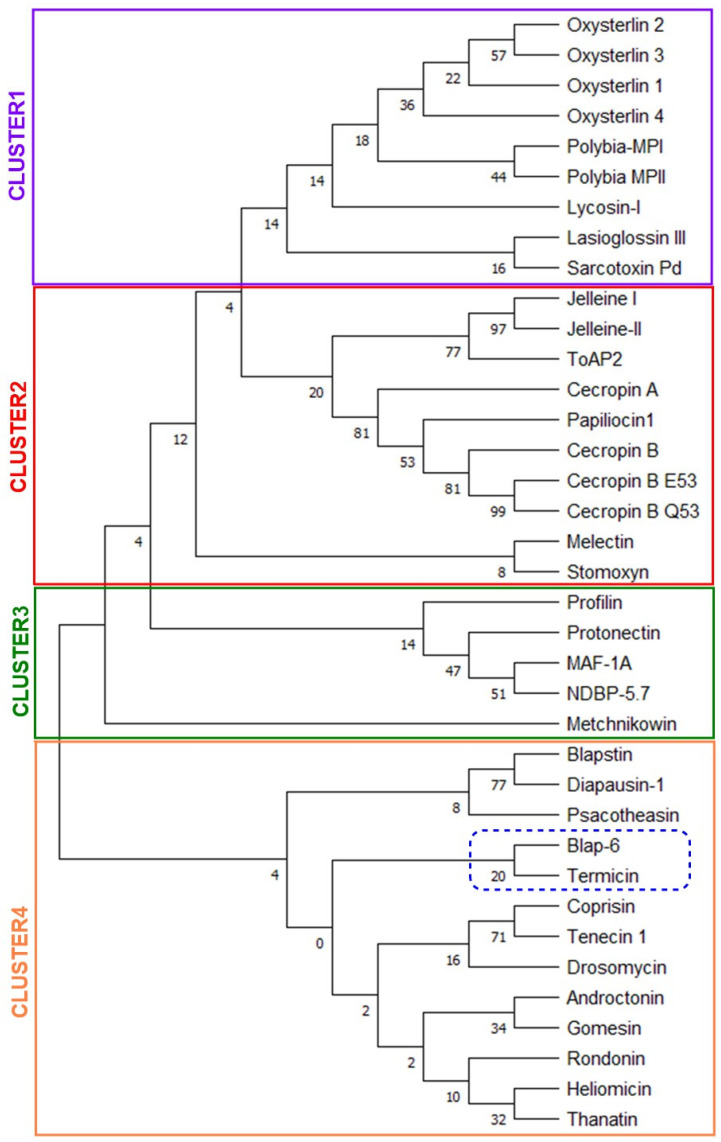
Phylogenetic tree (cladogram view) based on the amino acid sequences of insect peptides to determine their similarity or homologous character (to boost comprehension, it has been segmented into four clusters, each represented by a different color box).

This analysis offers several valuable benefits, particularly when identifying and optimizing therapeutic peptides. Using all thirty-seven-peptide sequences, we constructed the phylogenetic tree (Figure 5). Followed by pairwise and maximum likelihood distance between peptide sequences, the whole tree was separated into four clusters for better understanding, where we found that ‘Blap-6’ showed higher homologous with ‘termicin’ as it originated from same node and presented in cluster 4. In the same cluster, ‘blapstin with diapausin-1’, ‘coprisin with tenecin 1’, ‘androctonin with Gomesin’, and ‘heliomicin with thanatin’ were found as more homologous to each other. Similarly, in cluster-1, ‘lasioglossin-III with sarcotoxin Pd’, in cluster-2, ‘melectin with stomoxyn’, and in cluster-3, MAF-1A with NDBP-5.7 are considerably close to each other (Figure 5). Overall, the homologue-cum-phylogenetic tree analysis integrates evolutionary insights into peptide drug development, allowing for a more rational and effective approach to identifying, optimizing, and refining peptide therapeutics. This way, organizing peptides in different clusters and clades can be helpful in the future to determine if two peptides are very close or identical according to their sequence and why they show different properties. The cladogram comes to encourage further modification or conjugation to balance the activity and drug ability profiles for the higher translational success of peptides [37,38,39].

#### 2.1.6. Minimum Inhibitory Concentration and Minimum Fungicidal Concentration

The MIC is the lowest concentration of a peptide that inhibits the visible growth of a microorganism, and the MFC is the lowest concentration of a peptide resulting in the death of 50% of the inoculum. In this assay, the following concentrations of Blap-6 and Gomesin were tested: 300 and 200 mg/L; and 397 and 265 mg/L. The MIC that was observed by turbidity of wells achieved in this assay was 397 mg/L for Gomesin and 300 mg/L for Blap-6. All *Candida* spp. were eliminated (zero CFU/mL (MFC)) with all tested concentrations of both peptides. The MFC obtained by Gomesin was 397 mg/L, and that obtained by Blap-6 was 300 mg/L.

#### 2.1.7. Minimum Biofilm Eradication Concentration

The MBEC is the lowest concentration resulting in the death of 50% of the biofilm. The results showed the MBEC—but with 100% biofilm cell eradication—when using Gomesin at 265 mg/L for all *Candida* spp., except for *C. tropicalis* ATCC750 (minor but effective eradication) (Table 1). In general, it was noticed that the peptide Blap-6 (200 mg/L) allowed for inhibition of growth, but not as significantly as Gomesin, which had a greater performance in *Candida* spp. biofilms. Blap-6 was more effective against *C. albicans* SC5314 and less effective against *C. glabrata* ATCC2001. The MBEC for Gomesin was determined as follows: 265 mg/L for *C. albicans*, *C. glabrata*, and *C. parapsilosis*. For *C. tropicalis* (both peptides) and for Blap-6 (all *Candida* spp.), the MBEC was higher than those tested (Table 1).

The statistical analysis shows a *p*-value < 0.001 in comparison with the controls and Blap-6 for all *Candida* spp. and for the control of *C. tropicalis* and Gomesin, which means that both peptides are effective against the tested species but were not able to achieve complete eradication of biofilm. For *C. albicans*, *C. glabrata*, and *C. parapsilosis* with Gomesin, no significance was observed due to the complete eradication of biofilm, concluding that Gomesin is the most effective peptide against the tested *Candida* spp.

#### 2.1.8. Biomass Production Quantification

**Figure 6:**
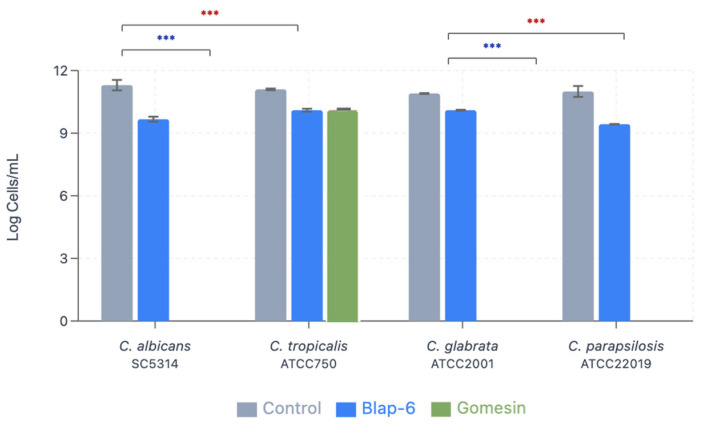
Minimum biofilm eradication concentration of *Candida* spp. after treatment with Blap-6 and Gomesin. Results presented as Log_10_ of cells/mL ± SD. *** indicates *p* < 0.001.

The biofilm biomass production was quantified with crystal violet staining with OD read at 570 nm (Table 2). For all *Candida* spp. species, Gomesin (265 mg/L) showed a reduction in biomass production between 16% and 49%. Interestingly, in contrast to the remaining assays, it was demonstrated that *Candida* spp. increased its biomass production in contact with Blap-6 (Figure 6). This increase suggests that this treatment may not inhibit biofilm growth and could even stimulate it, which was even more noticeable in *C. albicans* and *C. parapsilosis*.

As shown, Gomesin had a robust antifungal effect, with the greatest impact on *C. albicans* (−49%). The results also suggest that Gomesin is more effective against all tested species, with a marked reduction in its biomass.

#### 2.1.9. Confocal Laser Scanning Microscopy

**Figure 7:**
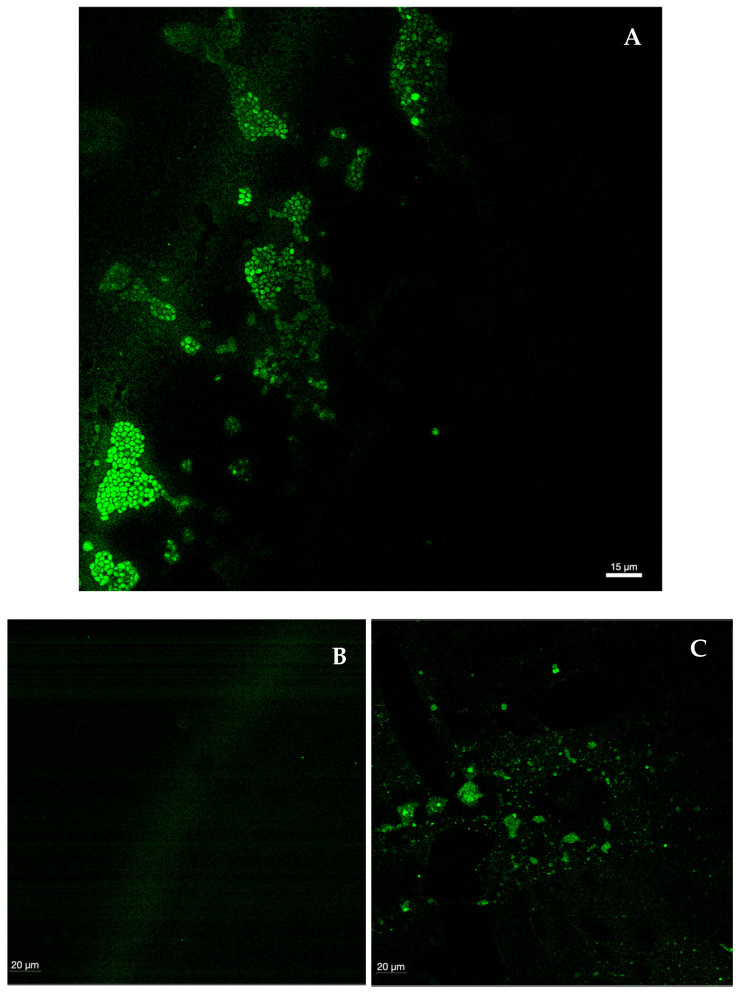

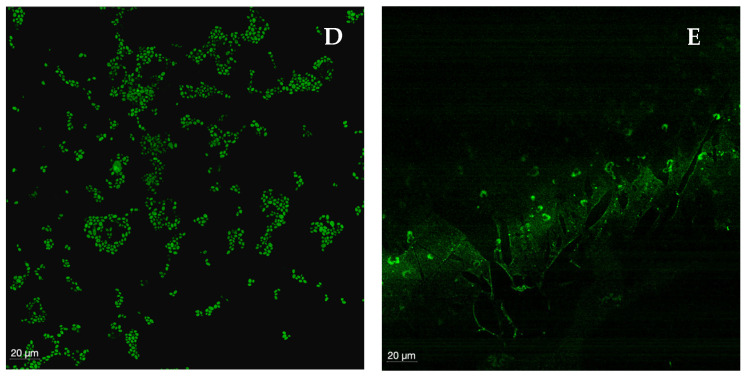
(**A**–**E**) Confocal laser scanning microscopy images of control of a 48 h biofilm of *Candida* (**A**), *C. albicans* SC5314 (**B**), *C. tropicalis* ATCC750 (**C**), *C. glabrata* ATCC2001 (**D**), and *C. parapsilosis* ATCC20019 (**E**) treated with Gomesin at 265 mg/L. The biofilm images were acquired using a confocal laser microscope (Leica Microsystems STELLARIS), and the images were treated with ImarisViewer version 10.2.0 and are at a magnification of 60×. Measure bar: 15 and 20 μm.

CLSM was used to visualize biofilms formed by each species, as well as their shape structure and thickness and to observe biofilms treated with Gomesin (the peptide with the best performance). In general, a marked difference in biofilm integrity was observed between the control and the biofilms treated with the peptide, though no linear or overlapping response was seen among all *Candida* species. The control (Figure 7A) showed clusters of intact, oval-shaped cells—consistent with the morphology of the genus *Candida*—as well as the extracellular matrix connecting these clusters. When comparing the four *Candida* species with the control, *C. albicans* exhibited the greatest loss of integrity, with only a minimal number of fully intact cells remaining. Predominantly, remnants of the matrix connecting the biofilm and intracellular contents were observed (Figure 7B). In *C. tropicalis*, small, sparse clusters of lysed cells and remnants of the biofilm extracellular matrix were present, though the destruction was less extensive than that observed in *C. albicans* (Figure 7C). The biofilm of *C. glabrata* was highly damaged, with visible “gaps” in the polysaccharide structure and very few viable cells (Figure 7D). In contrast, the biofilm of *C. parapsilosis* showed the highest number of cells among the treated biofilms, although still fewer and less aggregated than in the control. Notably, the polysaccharide matrix structure was not visible, indicating substantial destruction by the peptide (Figure 7E). In accordance with the MIC, MFC, and MBEC, Gomesin demonstrated a high anti-*Candida* and antibiofilm activity against all tested *Candida* species. In short, Gomesin was most effective against *C. albicans* and least effective against *C. parapsilosis*.

## 3. Discussion

*Candida* infections have become increasingly common worldwide and are also becoming more difficult to manage with conventional treatments, such as azoles and echinocandins [6,40,41]. Due to the emergence of resistant strains within each *Candida* species, it is essential to investigate and understand their resistance mechanisms and to identify potential new molecules capable of overcoming or at least partially surpassing these mechanisms. The development of new therapeutic alternatives aims to enhance treatment efficacy, thereby reducing the number of individuals exposed to these infections and, consequently, decreasing the development of resistance due to recurrent infections [41]. Because this microorganism is one of the four critical pathogens of the World Health Organization’s inaugural fungal priority pathogens list, the present study aimed to evaluate the potential of two insect-derived peptides, Blap-6 and Gomesin, as possible new molecules to be incorporated into future therapeutic strategies [42,43].

Exploring insect-derived antifungal peptides against *Candida* sp. presents a promising avenue for developing new therapeutic strategies to combat fungal infections, particularly in light of rising antifungal resistance [18,19,25]. This study provides comprehensive insights into the target-specific binding efficacy, drug chemistry, and possible molecular mechanisms of action of these peptides, reinforcing their potential as viable alternatives to conventional antifungals. Our findings demonstrate that insect-derived peptides exhibit significant binding affinities to key targets within *Candida* sp., in general, and *C. albicans* in particular, which is critical for its survival and pathogenicity. The detailed molecular docking analyses highlighted the specific interactions between these peptides and fungal targets, elucidating the mechanisms by which they exert their antifungal effects. By focusing on target specificity, we can develop therapeutic agents that minimize off-target effects and reduce the risk of adverse reactions in human cells, thereby enhancing patient safety. Molecular docking allows us to target specificity data that helps reduce risks to human cells by demonstrating binding selectivity between pathogen and human proteins, identifying potential cross-reactivity with human cell receptors, and revealing structural differences between microbial and human protein homologs [44]. The physicochemical properties of the insect-derived peptides were assessed to determine their drug-likeness and suitability for therapeutic applications. The favorable profiles observed in terms of solubility, stability, and permeability suggest that these peptides have the potential to be developed into effective antifungal agents. Understanding the drug chemistry aspects not only aids in optimizing the peptides but also facilitates the design of formulations that enhance bioavailability and efficacy. Particularly, in silico methods provided several significant advantages: computational approaches allowed for rapid screening and evaluation of a vast library of insect peptides, significantly reducing the time and costs associated with traditional laboratory methods [30,45]. In fact, this high-throughput capability enables researchers to identify promising candidates more quickly.

Our in silico studies provided detailed molecular insights that are often difficult to obtain through experimental methods alone. Here, we found that Blap-6 was the best candidate to be used as an antifungal compound for *Candida* sp. A few reports have also indicated interesting results when using (insect) peptides (natural or synthetic), showing potential biofilm inhibition in pathogenic fungal strains. Zhang et al. 2024 have described that Blap-6 is active against *Cryptococcus neoformans* [42]. Very interestingly, these authors have also verified that the peptide exhibited low hemolytic and cytotoxicity to human cells [46]. Schaefer et al. 2024 also explore the anti-*Candida* sp. effect of a novel and synthetic mimic positively charged peptide, similar to defensines, which also occur in insects, with very promising effects [47]. Another work, using a housefly larvae insect SVWC peptide 1 (WHIS1), has indicated that it inhibits *C. albicans* invasion into epithelial cells by affecting hyphal formation and adhesion gene expression [46]. Finally, Tancer et al., 2024, have evaluated a subunit of flippase, Cdc5. These are enzymes involved in moving phospholipids between the inner and outer layers of cell membranes, maintaining membrane asymmetry, which is crucial for cellular function, and they are also present in insects [48]. The authors evaluated cryptomycinamide (KKOO-NH_2_), derived from a nine-amino-acid segment of the *C. neoformans* Cdc50 protein, showing it to be a good option to be used in combination therapy with existing antifungal drugs [48]. In a theoretical perspective, Blap-6 could be used in combination therapies, leveraging its unique binding characteristics. While identifying the most suitable antifungal compounds would require experimental validation, the existing literature suggests that combinations of peptides with azoles (e.g., fluconazole) or echinocandins could enhance antifungal efficacy by targeting complementary pathways. Indeed, it is acknowledged that combination therapy can help reduce the development of resistance or re-sensitize resistant (*Candida* sp.) strains by targeting multiple pathways. It also lowers toxicity for the host by allowing for reduced drug concentrations. Inherently, antifungal peptides play a primary role in preventing and fighting fungal infections across all domains of life, mostly by interacting with fungal cell membranes, either damaging the cell wall or membrane directly or inducing intracellular stress [49,50,51,52].

We expanded the discussion to address hypothetical risks based on the physicochemical properties of Blap-6, such as immunogenicity (allergic reactions, formation of antibodies against the protein, potential cross-reactivity with other proteins), off-target effects (potential interaction with human proteins, effects on beneficial fungi in the human microbiome, possible disruption of normal flora), and potential toxicity (difference between therapeutic dose and toxic effects, accumulation in specific tissues), stability, pharmacokinetics, and delivery (oral intake? clearance rate from the body?). However, it has been shown that, in general, insect antifungal peptides exhibit broad-spectrum antifungal action with low drug resistance and few toxic and adverse consequences [42,53]. The phylogenetic tree analysis through computational platforms allowed for the refinement of peptide sequences to enhance their antifungal activity and their diversified relationships. This iterative process can lead to the design of more potent and selective peptides [30,39]. Predictive capability is critical for the development of long-lasting and effective treatments. In addition, in silico approaches can also assist in identifying potential synergies between insect peptides and existing antifungal agents, paving the way for combination therapies that may enhance treatment efficacy against resistant strains [30,39].

The results from our in vitro study demonstrate the significant antimicrobial activity of Blap-6 and Gomesin against *Candida* biofilms. In general, both Blap-6 and Gomesin showed highly significant statistical differences when compared with the controls (all conditions had a *p* < 0.001 ***). In particular, Gomesin completely eradicated (100%) three out of four *Candida* spp. biofilms, whereas with Blap-6, there was a significant reduction in biofilms (although with no complete eradication). *C. tropicalis* revealed to have some degree of peptide tolerance to both compounds when in biofilm form. This shows that Blap-6 and Gomesin are promising new molecules to fight against candidiasis, with Gomesin being more efficient, potentially due to its stable β-hairpin structure and optimized membrane-targeting properties. As shown by Rossi et al. (2012), Gomesin was effective against experimental *C. albicans* infections with MIC values of 5.5 μM for resistant strains [54]. An experimental study conducted by Chen et al. (2024) demonstrated the efficacy of another peptide extracted from an insect, WIHS1. When combined with a signal peptide, WIHS1 was effective in reducing the expression of genes responsible for biofilm formation as well as adhesion and invasion proteins of *C. albicans*. Additionally, this combination decreased the production of *C. albicans* hyphae. The structure of the signal peptide is highly similar to Gomesin, which also exhibited significant efficacy in eradicating *C. albicans* biofilm in our study [46]. Guevara-Lora et al. (2023) evaluated the antifungal activity of two synthetic peptides, ΔM3 and ΔM4, derived from cecropin D (an insect antimicrobial peptide). Both peptides exhibited an MFC > 99.9% against *C. albicans*, *C. tropicalis*, *C. glabrata*, and *C. parapsilosis*. These results are consistent with those obtained in our study, as we also observed an MFC > 99.9% for both peptides and species. Additionally, microscopy confirmed the degradation of the *Candida* spp. cell wall following peptide treatment, a finding also verified in our study, even with a different insect peptide. Notably, the biofilm production was inhibited, especially by the ΔM4 peptide in *C. albicans*. The molecular structure of ΔM4 is highly similar to Gomesin, which completely eradicated biofilm formation in *C. albicans* [55]. Studies by Galdiero et al. (2020) demonstrated that the gH625 peptide achieved similar MBEC values against *C. albicans* persister-derived biofilms, as well as our results [56]. The resistance of *C. tropicalis* observed in our study mirrors findings by Roscetto et al. (2018), who reported on VLL-28, showing that *C. tropicalis* was the most resistant in an MBEC assay [57]. Our results complement previous work by Alves et al. (2023) as well, who characterized *Candida* biofilm formation and antifungal susceptibility in clinical isolates, showing that biofilm-embedded cells demonstrate significantly reduced antimicrobial sensitivity [58]. The superior efficacy observed for the peptide Gomesin is also consistent with the reports of do Nascimento Dias et al. (2020), who demonstrated that peptides with β-hairpin structures exhibit greater antibiofilm activity compared to linear peptides [59]. This explains the significant difference in our results with Gomesin, which possesses a β-hairpin-like structure, in contrast to Blap-6, which has a linear structure. Our findings contribute to the growing body of evidence supporting that antimicrobial peptides are promising alternatives to conventional antifungals, particularly given the increasing prevalence of biofilm-associated resistance mechanisms. Finally, it is important to note that further assays are needed to confirm these results; more assays with a higher number of *Candida* strains/clinical isolates are needed, and more assays are needed to elucidate any tolerance/resistance mechanisms to both peptides.

Based on the results, we observed that the in silico prediction was accurate and played a crucial role in saving time that would have otherwise been spent analyzing less promising peptides. Furthermore, computational assays provide structural comparison capabilities, which are highly important for optimizing molecules to enhance their efficacy, as will be necessary for Blap-6. In summary, the exploration of insect-derived antifungal peptides through in silico methods not only underscores their potential as effective therapeutic agents against *Candida* sp. but also highlights the efficiency and advantages of computational approaches in drug discovery. As the incidence of fungal infections continues to rise and the efficacy of antifungals is dropping due to evolution of resistance, the insights gained from this study will inform future research aimed at developing innovative antifungal therapies, ultimately contributing to better clinical outcomes in the management of fungal diseases [30,39,45].

## 4. Materials and Methods

### 4.1. In Silico Analysis

#### 4.1.1. Antifungal Insect Peptides Library Report Selection

The insect-origin antifungal peptides were collected from the literature using a specific bibliographic search strategy from the PubMed, ScienceDirect, Wiley Library, Springer Online Library, and Taylor and Francis Online Library databases without any specific time limit during the publication search. An advanced search strategy was carried out based on keywords and MeSH (Medical Subject Headings) terms “*Candida*”, “biofilm”, “peptides”, and “insects” using Boolean operators. Finally, thirty-seven insect-derived peptides with antifungal activity against different fungal strains along with a recorded individual minimum inhibitory concentration (MIC) value were selected (Table S1).

#### 4.1.2. Three-Dimensional Peptide Structure Modelling and Molecular Docking Study

To explore the target-specific binding efficacy, the three-dimensional (3D) structure of the peptides as well as the desired target protein were required. Therefore, the de novo structure prediction computational framework PEP-FOLD 3.5 engine (https://bioserv.rpbs.univ-paris-diderot.fr/services/PEP-FOLD3/ (accessed on 26 November 2024)) was used to generate the theoretical structure from their peptide sequence [29,60]. Furthermore, the crystallographic 3D structure of the four most potent targeted enzymes of *C. albicans*, lanosterol 14-α-demethylase (LDM) (PDB ID: 5V5Z), secreted aspartic proteinase-5, or Sap-5 (PDB ID: 2QZX), N-myristoyl transferase (NMT) (PDB ID: 1NMT), and dihydrofolate reductase (DHFR) (PDB ID: 4HOF), was retrieved from the protein data bank (https://www.rcsb.org/ (accessed on 18 December 2024)). The peptide docking study was conducted using the HPEPDOCK (http://huanglab.phys.hust.edu.cn/hpepdock/ (accessed on 22 December 2024)) and HDOCK (http://hdock.phys.hust.edu.cn/ (accessed on 24 December 2024)) servers with default ab initio settings [29,61,62]. The software BIOVIA Discovery Studio Visualizer-2019 analyzed the structure of complex (protein–peptide) interactions after the docking analysis [29,63].

#### 4.1.3. Molecular Dynamics Simulation Study

The MD simulation was used to evaluate the structural stability of the lead peptide, Blap-6, in complex with LDM and to compare it with the standard antifungal drug fluconazole (FCZ). The LDM-Blap-6 and LDM-FCZ docking complexes were simulated for 100 ns using GROMACS 2022 with the AMBER99SB-ILDN force field [63,64]. Each complex was solvated in a TIP3P water box of ~103.6 nm^3^ and ~104.0 nm^3^, respectively [63,64]. The systems contained 34,192 and 34,563 water molecules. To neutralize the systems, 9 Cl^−^ ions were added to the peptide complex, while the FCZ system required no additional ions. Following energy minimization, both systems underwent 100 ps of NVT and NPT equilibration [63,64]. Production runs of 100 ns were performed, and structural stability was assessed via RMSD, RMSF, radius of gyration (Rg), and hydrogen bond analyses using standard GROMACS utilities [63,64].

#### 4.1.4. Physicochemical Property Analysis of Peptides

Physiochemical properties such as stability, solubility, and bioavailability are crucial parameters to determine the drug chemistry and drug ability profile of any peptide, thereby increasing the likelihood of experimental success [18,45]. Here, the tool ProtParam (https://web.expasy.org/protparam/ (accessed on 26 November 2024)) was used to record the basic properties of a peptide, including the aliphatic index (AI), grand average of hydropathicity (GRAVY), half-life value, instability index (II), total number of negatively charged (NC) residues (Asp+Glu), total number of positively charged (NP) residues (Arg+Lys), and theoretical isoelectric point prediction (pI) [45,65]. Note that the standard thresholds of GRAVY < 0 (indicating hydrophilicity) and instability index < 40 (suggesting protein stability) were used as benchmark criteria. Additionally, the water solubility characters of an amino acid were also defined by calculating the percentages of hydrophobicity (%Hy), acidity (%A), basicity (%B), and neutrality from the total (%N) using the PEPTIDE 2.0 (https://www.peptide2.com/ (accessed on 28 November 2024)) tool. The HeliQuest tool (https://heliquest.ipmc.cnrs.fr/ (accessed on 31 July 2025) visualized their 3D helical-wheel structural properties, including polarity and intramolecular bonding [29].

#### 4.1.5. Homologues-Cum-Phylogenetic Tree Analysis of Peptides

A phylogenetic tree was constructed to represent the evolutionary relationships among the thirty-seven insect peptides, illustrating how these peptides diverged from a common ancestor [29,66,67]. This evaluation offers insights into evolutionary patterns, structural adaptations, and functional changes across peptide species and genera. The tree was generated using Molecular Evolutionary Genetics Analysis (MEGA X, version 10.2.5) software (https://www.megasoftware.net/ (accessed on 28 December 2024)) following these specific procedures: statistical method—maximum likelihood (ML); test of phylogeny—bootstrapping with 500 replications; substitution model—Jones–Taylor–Thornton; rate and pattern—uniform rates; data subset treatment—used all sites without gaps or deletions; and tree inference in ML heuristic method—Nearest Neighbor Interchange with a strong branch swap filter and three processing threads [18,29].

### 4.2. In Vitro Assays

#### 4.2.1. Organisms and Growth Conditions

The *Candida* isolates used in this study were *Candida albicans* SC5314, *Candida tropicalis* ATCC750, *Candida glabrata* ATCC2001, and *Candida parapsilosis* ATCC20019, from the American Type Culture Collection (ATCC) (Manassas, VA, USA).

All species were pre-subcultured on Sabouraud Dextrose Agar (SDA) medium and/or Sabouraud Dextrose Broth (SBD) (Merck, Darmstadt, Germany). The subcultured plates were incubated at 37 °C for 24 h. Aliquots were stored at −80 °C with SDB and 20% glycerol.

#### 4.2.2. Insect Peptides

Blap-6 and Gomesin were selected to perform the following assays. Both peptides were synthesized and kindly provided by PepMic Co., Ltd. (Suzhou, China). Blap-6 was previously isolated from *Blaps rhychopetera* beetle and has a 17 amino acid sequence of KRCRFRIYRWGFPRRRF [42]. Gomesin was isolated from *Acanthoscurria gomesiana* spider and has an 18 amino acid sequence of ZCRRLCYKQRCVTYCRGR (produced with no “Z”) [42]. Both lyophilized peptides were dissolved with sterile ultrapure water at 794 mg/L and 600 mg/L stock solution, respectively, and stored at −18 °C until use. The tested concentrations were selected according to previous reports: Blap-6—794 mg/L, 397 mg/L, and 265 mg/L, and Gomesin—600 mg/L, 300 mg/L, and 200 mg/L.

#### 4.2.3. Minimum Inhibitory Concentration and Minimum Fungicidal Concentration

The minimum inhibitory concentration (MIC) was assessed using the microdilution method and according to the EUCAST guidelines [68]. Blap-6 was tested at concentrations of 300 mg/L and 200 mg/L and Gomesin at concentrations of 397 mg/L and 265 mg/L. This assay was repeated a second time to confirm the results. Fresh colonies of each species were picked from SDA plates and suspended with sterile water to reach 0.5 McFarland turbidity, followed by a 1/10 dilution with RPMI-1640 (PAN-Biotech, Aidenbach, Germany). An aliquot of 100 μL from each *Candida* spp. was added to 96-well polystyrene microtiter plates (Wuxi NEST Biotechnology Co., Ltd., Wuxi, China), and an equal volume of each peptide was added (total volume = 200 μL). Positive controls with *Candida* spp. suspensions in RPMI-1640 and negative controls with RPMI-1640 only were also included for each species. All plates were incubated at 37 °C for 24 h. The plates were analyzed by naked eye, according to the guidelines [68,69].

After incubation, the minimum fungicidal concentration (MFC) was determined. The MFC was defined as the lowest peptide concentration capable of killing 50% of the initial inoculum. For that, serial decimal dilutions with Phosphate-Buffered Saline (PBS, pH = 7, 0.1 M) (0 to −8) were performed and plated on SDA and incubated at 37 °C for 24 h. After 24 h, the number of CFUs formed was determined [69,70].

#### 4.2.4. Minimum Biofilm Eradication Concentration

The minimum biofilm eradication concentration (MBEC) was assessed by the microdilution method, adapted from the EUCAST guidelines, and defined as the lowest peptide concentration capable of killing 50% of the biofilm cells [69]. According to the previous results (MIC and MFC), Blap-6 was tested at a concentration of 200 mg/L and Gomesin at a concentration of 265 mg/L.

For this, 200 μL of cell suspensions from each *Candida* spp. in this study were placed in 96-well polystyrene microtiter plates. After 24 h of incubation at 37 °C and 120 rpm, 100 μL of RPMI-1640 was removed and replaced with another 100 μL of peptides (Blap-6: 200 mg/L; Gomesin: 265 mg/L—2× concentrated) or fresh RPMI-1640 (positive control) and incubated at 37 °C and 120 rpm for another 24 h (total: 48 h, mature biofilms). Then, all medium was removed from the wells, and the biofilms were carefully washed with 200 μL of PBS to remove planktonic cells. Next, biofilms were scraped, and the formed suspensions were transferred to another 96-well plate to carry out serial decimal dilutions, plated on SDA, and finally incubated at 37 °C for 24 h. The CFUs were counted, and the results were presented as percentage of biofilm cell reduction [70].

#### 4.2.5. Biomass Production Quantification

Crystal violet (CV) staining was used to quantify biomass production by *Candida* species treated with peptides. After 48 h of biofilm formation, all medium was removed from the wells, and 200 μL of methanol was added to fix the formed biofilm for 15 min. After air-drying, biofilms were stained with 200 μL of CV (1% *v*/*v*) for 5 min. Following the CV removal and a double rinse with sterile water, the stain was dissolved by adding 200 μL of 33% (*v*/*v*) of acetic acid. Finally, absorbance readings of the obtained solutions were measured at 570 nm using a microtiter plate reader. The results were presented as % of biofilm reduction (compared with the controls) [69].

#### 4.2.6. Confocal Laser Scanning Microscopy (CLSM)

Biofilms were prepared on coupons placed inside 6-well polystyrene cell culture plates (Wuxi NEST Biotechnology Co., Ltd., Wuxi, China). A total of 1000 μL of each *Candida* spp. suspension (1 × 10^5^ cells/mL in RPMI-1640) was added to each well. The plates were incubated for 24 h at 37 °C with agitation at 120 rpm. After 24 h, 500 μL of medium was removed and replaced with another 500 μL of RPMI-1640 (positive control) or 500 μL of RPMI-1640 with peptide (265 mg/L). The plates were incubated for another 24 h. Biofilms were fixed with 100% (*v*/*v*) methanol for 20 min, followed by 4% (*w*/*v*) of paraformaldehyde (Sigma-Aldrich, St. Louis, MO, USA) 15 min and then 50% (*v*/*v*) ethanol. Then, the plates were allowed to air-dry [71].

To stain *Candida* spp. biofilm cells, a specific 23S rRNA PNA probe developed and optimized by our group was used for *Candida* spp. detection: 5′-Alexa488-OO-CACCCACAAAATCAA-3′ (melting temperature: 75.69 °C; specificity: 96.04%; sensibility: 84.79%). The probe was synthesized (Panagene, Daejoen, Republic of Korea), attached to the Alexa^®^-488 fluorochrome device, and tested with *Candida albicans* SC5314. Briefly, the biofilms were formed on 6-well microtiter plates. After, the biofilms were washed with saline solution to remove loosely bound cells and then placed in a Petri dish to dry at 60 °C for 15 min. After that, they were fixed with 100% methanol for 20 min, followed by 4% paraformaldehyde for 15 min. Thereafter, they were air-dried on the bench until fully dry. After this, the FISH procedure was applied. For that, the biofilms were incubated with PNA Probe at 54 °C for 30 min in the dark. The samples were observed on a STELLARIS 5 device mounted on a Leica DM6 B upright microscope (Leica Microsystems, Wetzlar, Germany), equipped with White-Light Laser (WLL) as an excitation light source (from 485 to 685 nm) and 2 internal detection channels equipped with Power HyD S. Image acquisition was performed using a 63× oil-immersion objective (63×/1.4 W) and the 488 nm laser line. Z-stacks with 1 µm Z-steps were collected. All microscope settings were identical among the analyzed groups. The Zeiss Zen software (version 10.0.2) was used for confocal image acquisition and processing.

### 4.3. Statistical Analysis

All experiments were performed in duplicates and in two independent assays. The comparation of results was based on two-sample *t*-tests with equal variances to compare treatment groups with respective controls within each *Candida* species. The software used for statistical analysis was GraphPad Prism 7 (GraphPad Software, San Diego, CA, USA). Statistical significance was set at *p* < 0.05, which leads to a confidence level of 95%. All calculated t-values exceeded the critical value (t_0.05,2_ = 4.303), indicating *p* < 0.001 for all comparisons where statistical testing was applicable.

## 5. Conclusions

This study highlights the significant potential of insect-derived antifungal peptides as promising candidates for combating candidiasis. Through in-depth computer studies, we showed that they could bind to their targets, and this helped us understand the possible molecular mechanisms that we can target to stop the infection. The molecular docking studies showed different binding affinities, which suggests that Blap-6 can effectively inhibit key cellular functions in *Candida* sp. Furthermore, the MD simulation results for Blap-6 in comparison with a standard antifungal drug, FCZ, against LDM showed quite similar drug stability, and along with their physicochemical properties, this indicates a favorable drug-like profile, supporting their viability as therapeutic agents. Our results also show the importance of using computers early on in the drug discovery process, as this approach allows for quick detection and for improving peptide candidates while using as few resources as possible. Through wet lab experiments, we turned the provisions obtained by the computer studies valuable, which proved that Blap-6 and Gomesin have marked efficacy against *Candida albicans* and NCACs infections. More studies are needed to enhance the potential of Blap-6, mainly in *C. tropicalis*. By addressing the pressing challenge of antifungal resistance, this study contributes to the broader effort to develop innovative antifungal therapies that are both effective and selective, ultimately paving the way for improved clinical outcomes in the treatment of fungal infections. In the future, researchers should focus on testing the most promising peptides found in this study and seeing if they can be used in combination therapies to make them even more effective against strains of *Candida* sp. resistant to common antifungal treatments.

## Figures and Tables

**Table 1:** Log_10_ of cells/mL ± standard deviation (SD) of biofilm *Candida* cells using MBEC tested for Blap.6 200 mg/L and Gomesin 265 mg/L. Note: Values represent mean + standard deviation from *n* = 2 independent experiments performed in duplicates. Statistical comparisons show control vs. treatment for each species individually.

	Log Cells/mL ± SD		
	Control	Blap-6 (200 mg/L)	Gomesin (265 mg/L)
*C. albicans* SC5314	11.30 ± 0.25	9.67 ± 0.12 (14.4% reduction)	0.00 ± 0.00 (100% reduction) Complete eradication
*C. tropicalis* ATCC750	11.10 ± 0.04	10.10 ± 0.07 (9.0% reduction)	10.10 ± 0.01 (9.0% reduction)
*C. glabrata* ATCC2001	10.90 ± 0.02	10.10 ± 0.02 (7.3% reduction)	0.00 ± 0.00 (100% reduction) Complete eradication
*C. parapsilosis* ATCC20019	11.00 ± 0.26	9.43 ± 0.02 (14.3% reduction)	0.00 ± 0.00 (100% reduction) Complete eradication

**Table 2:** Percentage of biomass production in *C. albicans* SC5314, *C. tropicalis* ATCC750, *C. glabrata* ATCC2001, and *C. parapsilosis* ATCC20019 after treatment with Blap-6 (200 mg/L) and Gomesin (265 mg/L).

	% Biomass	
	Blap-6 (200 mg/L)	Gomesin (265 mg/L)
*C. albicans* SC5314	+47%	−49%
*C. tropicalis* ATCC750	+21%	−35%
*C. glabrata* ATCC2001	+16%	−19%
*C. parapsilosis* ATCC20019	+46%	−16%

## Data Availability

Data is contained within the article and Supplementary Materials.

## Supplementary Materials

The supporting information can be downloaded at: https://www.mdpi.com/article/10.3390/ijms26157449/s1.

## References

[1] Lopes J.P., Lionakis M.S. (2022). Pathogenesis and Virulence of Candida Albicans. Virulence.

[2] d’Enfert C., Janbon G. (2016). Biofilm formation in Candida glabrata: What have we learnt from functional genomics approaches?. FEMS Yeast Res..

[3] Cavalheiro M., Teixeira M.C. (2018). Candida Biofilms: Threats, Challenges, and Promising Strategies. Front. Med..

[4] Pappas P.G., Lionakis M.S., Arendrup M.C., Ostrosky-Zeichner L., Kullberg B.J. (2018). Invasive Candidiasis. Nat. Rev. Dis. Primers.

[5] Soriano A., Honore P.M., Puerta-Alcalde P., Garcia-Vidal C., Pagotto A., Gonçalves-Bradley D.C., Verweij P.E. (2023). Invasive Candidiasis: Current Clinical Challenges and Unmet Needs in Adult Populations. J. Antimicrob. Chemother..

[6] Lass-Flörl C., Kanj S.S., Govender N.P., Thompson G.R., Ostrosky-Zeichner L., Govrins M.A. (2024). Invasive Candidiasis. Nat. Rev. Dis. Primers.

[7] WHO (2022). WHO Fungal Pathogens Priority List.

[8] Beardsley J., Halliday C.L., Chen S.C.A., Sorrell T.C. (2018). Responding to the Emergence of Antifungal Drug Resistance: Perspectives from the Bench and the Bedside. Future Microbiol..

[9] Logan A., Wolfe A., Williamson J.C. (2022). Antifungal Resistance and the Role of New Therapeutic Agents. Curr. Infect. Dis. Rep..

[10] De Oliveira H.C., Bezerra B.T., Rodrigues M.L. (2022). Antifungal Development and the Urgency of Minimizing the Impact of Fungal Diseases on Public Health. ACS Bio Med. Chem. Au.

[11] Pereira R., dos Santos Fontenelle R.O., de Brito E.H.S., de Morais S.M. (2021). Biofilm of Candida Albicans: Formation, Regulation and Resistance. J. Appl. Microbiol..

[12] Gulati M., Nobile C.J. (2016). Candida Albicans Biofilms: Development, Regulation, and Molecular Mechanisms. Microbes Infect..

[13] Cui X., Wang L., Lü Y., Yue C. (2022). Development and Research Progress of Anti-Drug Resistant Fungal Drugs. J. Infect. Public Health.

[14] Augostine C.R., Avery S.V. (2022). Discovery of Natural Products with Antifungal Potential Through Combinatorial Synergy. Front. Microbiol..

[15] Shariati A., Didehdar M., Razavi S., Heidary M., Soroush F., Chegini Z. (2022). Natural Compounds: A Hopeful Promise as an Antibiofilm Agent Against Candida Species. Front. Pharmacol..

[16] Roe K. (2023). Treatment Alternatives for Multidrug-Resistant Fungal Pathogens. Drug Discov. Today.

[17] Najafian L., Babji A.S. (2012). A Review of Fish-Derived Antioxidant and Antimicrobial Peptides: Their Production, Assessment, and Applications. Peptides.

[18] Stączek S., Cytryńska M., Zdybicka-Barabas A. (2023). Unraveling the Role of Antimicrobial Peptides in Insects. Int. J. Mol. Sci..

[19] Zhang Q.Y., Yan Z.B., Meng Y.M., Hong X.Y., Shao G., Ma J.J., Cheng X.R., Liu J., Kang J., Fu C.Y. (2021). Antimicrobial Peptides: Mechanism of Action, Activity and Clinical Potential. Mil. Med. Res..

[20] Ahmed I., Asgher M., Sher F., Hussain S.M., Nazish N., Joshi N., Sharma A., Parra-Saldívar R., Bilal M., Iqbal H.M.N. (2022). Exploring Marine as a Rich Source of Bioactive Peptides: Challenges and Opportunities from Marine Pharmacology. Mar. Drugs.

[21] Lima A.M., Azevedo M.I.G., Sousa L.M., Oliveira N.S., Andrade C.R., Freitas C.D.T., Souza P.F.N. (2022). Plant Antimicrobial Peptides: An Overview about Classification, Toxicity and Clinical Applications. Int. J. Biol. Macromol..

[22] Guryanova S.V., Balandin S.V., Belogurova-Ovchinnikova O.Y., Ovchinnikova T.V. (2023). Marine Invertebrate Antimicrobial Peptides and Their Potential as Novel Peptide Antibiotics. Mar. Drugs.

[23] Ganeshkumar A., Gonçale J.C., Rajaram R., Junqueira J.C. (2023). Anti-Candidal Marine Natural Products: A Review. J. Fungi.

[24] Shai Y., Makovitzky A., Avrahami D., Makovitzki A. (2006). Host Defense Peptides and Lipopeptides: Modes of Action and Potential Candidates for the Treatment of Bacterial and Fungal Infections. Curr. Protein Pept. Sci..

[25] Prusty J.S., Kumar A., Kumar A. (2024). Anti-Fungal Peptides: An Emerging Category with Enthralling Therapeutic Prospects in the Treatment of Candidiasis. Crit. Rev. Microbiol..

[26] Zida A., Bamba S., Yacouba A., Ouedraogo-Traore R., Guiguemdé R.T. (2017). Anti-Candida Albicans Natural Products, Sources of New Antifungal Drugs: A Review. J. Mycol. Med..

[27] Rodríguez-Castaño G.P., Rosenau F., Ständker L., Firacative C. (2023). Antimicrobial Peptides: Avant-Garde Antifungal Agents to Fight against Medically Important Candida Species. Pharmaceutics.

[28] Ramazi S., Mohammadi N., Allahverdi A., Khalili E., Abdolmaleki P. (2022). A Review on Antimicrobial Peptides Databases and the Computational Tools. Database.

[29] Sahoo A., Swain S.S., Panda S.K., Hussain T., Panda M., Rodrigues C.F. (2022). In Silico Identification of Potential Insect Peptides against Biofilm-Producing Staphylococcus Aureus. Chem. Biodivers..

[30] Sama-ae I., Pattaranggoon N.C., Tedasen A. (2023). In Silico Prediction of Antifungal Compounds from Natural Sources towards Lanosterol 14-Alpha Demethylase (CYP51) Using Molecular Docking and Molecular Dynamic Simulation. J. Mol. Graph. Model..

[31] Woodburn K.W., Edward Clemens L., Jaynes J., Joubert L.M., Botha A., Nazik H., Stevens D.A. (2019). Designed Antimicrobial Peptides for Recurrent Vulvovaginal Candidiasis Treatment. Antimicrob. Agents Chemother..

[32] Datta A., Ghosh A., Airoldi C., Sperandeo P., Mroue K.H., Jimenez-Barbero J., Kundu P., Ramamoorthy A., Bhunia A. (2015). Antimicrobial Peptides: Insights into Membrane Permeabilization, Lipopolysaccharide Fragmentation and Application in Plant Disease Control. Sci. Rep..

[33] Swain S.S., Singh S.R., Sahoo A., Panda P.K., Hussain T., Pati S. (2022). Integrated Bioinformatics-Cheminformatics Approach toward Locating Pseudo-Potential Antiviral Marine Alkaloids against SARS-CoV-2-Mpro. Proteins.

[34] Zomorodbakhsh S., Abbasian Y., Naghinejad M., Sheikhpour M. (2020). The Effects Study of Isoniazid Conjugated Multi-Wall Carbon Nanotubes Nanofluid on *Mycobacterium tuberculosis*. Int. J. Nanomed..

[35] Wang L., Wang N., Zhang W., Cheng X., Yan Z., Shao G., Wang X., Wang R., Fu C. (2022). Therapeutic Peptides: Current Applications and Future Directions. Signal Transduct. Target Ther..

[36] Lee M.F., Poh C.L. (2023). Strategies to Improve the Physicochemical Properties of Peptide-Based Drugs. Pharm. Res..

[37] Fuchs J.E., Wellenzohn B., Weskamp N., Liedl K.R. (2015). Matched Peptides: Tuning Matched Molecular Pair Analysis for Biopharmaceutical Applications. J. Chem. Inf. Model..

[38] Saleem A., Rajput S. (2020). Insights from the in Silico Structural, Functional and Phylogenetic Characterization of Canine Lysyl Oxidase Protein. J. Genet. Eng. Biotechnol..

[39] Cao W., Wu L.Y., Xia X.Y., Chen X., Wang Z.X., Pan X.M. (2023). A Sequence-Based Evolutionary Distance Method for Phylogenetic Analysis of Highly Divergent Proteins. Sci. Rep..

[40] Kumar S., Kumar A., Roudbary M., Mohammadi R., Černáková L., Rodrigues C.F. (2022). Overview on the Infections Related to Rare Candida Species. Pathogens.

[41] Rocha W.R.V.d., Nunes L.E., Neves M.L.R., Ximenes E.C.P.d.A., Albuquerque M.C.P.d.A. (2021). Candida Genus—Virulence Factores, Epidemiology, Candidiasis and Resistance Mechanisms. Res. Soc. Dev..

[42] Zhang L.M., Zhou S.W., Huang X.S., Chen Y.F., Mwangi J., Fang Y.Q., Du T., Zhao M., Shi L., Lu Q.M. (2024). Blap-6, a Novel Antifungal Peptide from the Chinese Medicinal Beetle Blaps Rhynchopetera against Cryptococcus Neoformans. Int. J. Mol. Sci..

[43] WHO Fungal Priority Pathogens List to Guide Research, Development and Public Health Action. https://www.who.int/publications/i/item/9789240060241.

[44] Odchimar N.M.O., Dulay A.N.G., Orosco F.L. (2025). Molecular Modelling and Optimization of a High-Affinity Nanobody Targeting the Nipah Virus Fusion Protein through in Silico Site-Directed Mutagenesis. Comput. Biol. Chem..

[45] Sahoo A., Swain S.S., Paital B., Panda M. (2022). Combinatorial Approach of Vitamin C Derivative and Anti-HIV Drug-Darunavir against SARS-CoV-2. Front. Biosci. (Landmark Ed.).

[46] Chen M., Huang W.K., Yao Y., Wu S.M., Yang Y.X., Liu W.X., Luo G., Wei S.F., Zhang H., Liu H.M. (2024). Heterologous Expression of the Insect SVWC Peptide WHIS1 Inhibits Candida Albicans Invasion into A549 and HeLa Epithelial Cells. Front. Microbiol..

[47] Schaefer S., Vij R., Sprague J.L., Austermeier S., Dinh H., Judzewitsch P.R., Müller-Loennies S., Lopes Silva T., Seemann E., Qualmann B. (2024). A Synthetic Peptide Mimic Kills Candida Albicans and Synergistically Prevents Infection. Nat. Commun..

[48] Tancer R.J., Pawar S., Wang Y., Ventura C.R., Wiedman G., Xue C. (2024). Improved Broad Spectrum Antifungal Drug Synergies with Cryptomycin, a Cdc50-Inspired Antifungal Peptide. ACS Infect. Dis..

[49] Spitzer M., Robbins N., Wright G.D. (2017). Combinatorial Strategies for Combating Invasive Fungal Infections. Virulence.

[50] Revie N.M., Iyer K.R., Maxson M.E., Zhang J., Yan S., Fernandes C.M., Meyer K.J., Chen X., Skulska I., Fogal M. (2022). Targeting Fungal Membrane Homeostasis with Imidazopyrazoindoles Impairs Azole Resistance and Biofilm Formation. Nat. Commun..

[51] dos Reis T.F., de Castro P.A., Bastos R.W., Pinzan C.F., Souza P.F.N., Ackloo S., Hossain M.A., Drewry D.H., Alkhazraji S., Ibrahim A.S. (2023). A Host Defense Peptide Mimetic, Brilacidin, Potentiates Caspofungin Antifungal Activity against Human Pathogenic Fungi. Nat. Commun..

[52] de Ullivarri M.F., Arbulu S., Garcia-Gutierrez E., Cotter P.D. (2020). Antifungal Peptides as Therapeutic Agents. Front. Cell. Infect. Microbiol..

[53] Ratcliffe N.A., Mello C.B., Garcia E.S., Butt T.M., Azambuja P. (2011). Insect Natural Products and Processes: New Treatments for Human Disease. Insect Biochem. Mol. Biol..

[54] Rossi D.C., Muñoz J.E., Carvalho D.D., Belmonte R., Faintuch B., Borelli P., Miranda A., Taborda C.P., Daffre S. (2012). Therapeutic Use of a Cationic Antimicrobial Peptide from the Spider Acanthoscurria Gomesiana in the Control of Experimental Candidiasis. BMC Microbiol..

[55] Guevara-Lora I., Bras G., Juszczak M., Karkowska-Kuleta J., Gorecki A., Manrique-Moreno M., Dymek J., Pyza E., Kozik A., Rapala-Kozik M. (2023). Cecropin D-Derived Synthetic Peptides in the Fight against Candida Albicans Cell Filamentation and Biofilm Formation. Front. Microbiol..

[56] Galdiero E., de Alteriis E., De Natale A., D’Alterio A., Siciliano A., Guida M., Lombardi L., Falanga A., Galdiero S. (2020). Eradication of Candida Albicans Persister Cell Biofilm by the Membranotropic Peptide GH625. Sci. Rep..

[57] Roscetto E., Contursi P., Vollaro A., Fusco S., Notomista E., Catania M.R. (2018). Antifungal and Anti-Biofilm Activity of the First Cryptic Antimicrobial Peptide from an Archaeal Protein against Candida Spp. Clinical Isolates. Sci. Rep..

[58] Alves A.M.C.V., Lopes B.O., Leite A.C.R.d.M., Cruz G.S., Brito É.H.S.d., Lima L.F.d., Černáková L., Azevedo N.F., Rodrigues C.F. (2023). Characterization of Oral Candida Spp. Biofilms in Children and Adults Carriers from Eastern Europe and South America. Antibiotics.

[59] do Nascimento Dias J., de Souza Silva C., de Araújo A.R., Souza J.M.T., de Holanda Veloso Júnior P.H., Cabral W.F., da Glória da Silva M., Eaton P., de Souza de Almeida Leite J.R., Nicola A.M. (2020). Mechanisms of Action of Antimicrobial Peptides ToAP2 and NDBP-5.7 against Candida Albicans Planktonic and Biofilm Cells. Sci. Rep..

[60] Lamiable A., Thevenet P., Rey J., Vavrusa M., Derreumaux P., Tuffery P. (2016). PEP-FOLD3: Faster de Novo Structure Prediction for Linear Peptides in Solution and in Complex. Nucleic Acids Res..

[61] Zhou P., Jin B., Li H., Huang S.Y. (2018). HPEPDOCK: A Web Server for Blind Peptide-Protein Docking Based on a Hierarchical Algorithm. Nucleic Acids Res..

[62] Yan Y., Tao H., He J., Huang S.Y. (2020). The HDOCK Server for Integrated Protein–Protein Docking. Nat. Protoc..

[63] Swain S.S., Sahoo A., Singh S.R., Sahoo J., Paidesetty S.K. (2024). Synthesis, Spectroscopic Analysis, and Computational-Based Investigations on “azo-Coumarin-Co(II)-Galangin” Hybrids Exhibit Multipotential Activities. J. Biomol. Struct. Dyn..

[64] Sahoo A., Paidesetty S.K., Panda M. (2025). Target-Specific High-Throughput Screening of Anti-Inflammatory Phytosteroids for Autoimmune Diseases: A Molecular Docking-Dynamics Simulation Approach. Steroids.

[65] Gasteiger E., Gattiker A., Hoogland C., Ivanyi I., Appel R.D., Bairoch A. (2003). ExPASy: The Proteomics Server for in-Depth Protein Knowledge and Analysis. Nucleic Acids Res..

[66] Zaman W., Ye J., Saqib S., Liu Y., Shan Z., Hao D., Chen Z., Xiao P. (2021). Predicting Potential Medicinal Plants with Phylogenetic Topology: Inspiration from the Research of Traditional Chinese Medicine. J. Ethnopharmacol..

[67] Chase K., Watkins M., Safavi-Hemami H., Olivera B.M. (2022). Integrating Venom Peptide Libraries Into a Phylogenetic and Broader Biological Framework. Front. Mol. Biosci..

[68] EUCAST (2003). Definitive Document, E.Def 7.4. Method for the Determination of Broth Dilution Minimum Inhibitory Concentrations of Antifungal Agents for Yeasts.

[69] Maziere M., Rompante P., Andrade J.C., De Oliveira B.S.F., Alves M.C., Rodrigues C.F. (2025). Repurposing Mouthwashes: Antifungal and Antibiofilm Abilities of Commercially Available Mouthwashes Against *Candida* spp.. Antibiotics.

[70] Rajão A., Silva J.P.N., Almeida-Nunes D.L., Rompante P., Rodrigues C.F., Andrade J.C. (2025). Limosilactobacillus Reuteri AJCR4: A Potential Probiotic in the Fight Against Oral Candida Spp. Biofilms. Int. J. Mol. Sci..

[71] Rodrigues C.F., Boas D.V., Haynes K., Henriques M. (2018). The MNN2 Gene Knockout Modulates the Antifungal Resistance of Biofilms of Candida Glabrata. Biomolecules.

