# Supplementary material for: In Silico and In Vitro Potential Antifungal Insights of Insect-Derived Peptides in the Management of Candida sp. Infections
Source: Int J Mol Sci. 2025 Aug 1;26(15):7449. doi: 10.3390/ijms26157449 (PMC12347408; doi:10.3390/ijms26157449)
Supplement: Supplementary file 1 [file ijms-26-07449-s001.zip › ijms-3659429-supplementary.pdf]

Supplementary data

Table S1. List of insect-derived peptides having antifungal activity against different pathogenic fungal strains along with more relevant information.

Sl. No	Insect peptide (Source of insect)	Size of peptide (Sequence of the peptide)	MFC (µM/µg/mL)	Tested fungal strains	References
1	Androctonin (*Androctonus australis*)	25(RSVCRQIKICRRRGGCYYKCTNRPY)	1.6–3.15 µM	*C. albicans*	Hetru et al., 2000; Silva et al., 2000
2	Blap-6 (*Blaps rhynchopetera*)	17 (KRCRFRIYRWGFPRRRF)	1.22 µM<br>0.81 µM	*C. albicans*<br>*C. neoformans*	Zhang et al., 2024
3	Blapstin (*Blaps rhynchopetera*)	41(VRVGPCDQVCSRTNPEKDECCRAHGHSGHSSCYGGRMNCYG)	3.5 µM<br>5.3 µM<br>7 µM	*C. albicans* 0065<br>*Trichophyton rubrum*<br>*C. albicans* ATCC 10231<br>*C. albicans* 0063 & 6	Zhang et al., 2023
4	Cecropin A (*D. melanogaster* and *H. cecropia*)	34(RWKLFKKIEKVGRNVRDGLIKAGPAIAVIGQAKSLGK)	0.6 to 2.5 µg/mL<br>12.4 µg/mL	*C. albicans*<br>*F. oxysporum*	Andra et al., 2001; Mirza et al., 2018
5	Cecropin B (*D. melanogaster* and *H. cecropia*)	34(KWKVFKKIEKMGRNIRNGIVKAGPAIAVLGEAKAL)	0.6 to 1.3 µM<br>9.5 µM	*C. albicans*<br>*A. fumigatus*	Andra et al., 2001; Mirza et al., 2018
6	Cecropin B_E53 (*Bombyx mori*)	35(RWKIFKKIEKMGRNIRDGIVKAGPAIEVLGSAKAI)	>20 µM	*C. albicans*	Romoliet al., 2019
7	Cecropin B_Q53 (*Bombyx mori*)	35(RWKIFKKIEKMGRNIRDGIVKAGPAIQVLGSAKAI)	>20 µM	*C. albicans*	Romoliet al., 2019
8	Coprisin (*Copris tripartitus*)	43(VTCDVLSFEAKGIAVNHSACALHCIALRKKGGSCQNGVCVCRN)	5-20 µM	*A. flavus, A. fumigatus*<br>*A. parasiticus, C. albicans*<br>*C. parapsilosis, M. furfur*<br>*T. beigelii, & T. rubrum*	Lee et al., 2012
9	Diapausin-1 (*Manduca sexta*)	45(INNWVRVPPCDQVCSRSNPEKDECCRAHGHAFHAHCNGGMNCYRR)	12 µM	*S. cerevisiae*	Al Souhail et al., 2016
10	Drosomycin (*Podisus maculiveris* and *D. melanogaster*)	44(DCLSGRYKGPCAVWDNETCRRVCKEEGRSSGHCSPSLKCWCEGC)	0.30-0.75 µM<br><br>5.9-12.3 µM	*N. crassa, G. candidum,* &<br>*S. cerevisiae*<br>*F. oxysporum*	Tian et al., 2020; Mirza et al., 2018
11	Gomesin (*Acanthoscurria gomesiana*)	18(ZCRRLCYKQRCVTYCRGR)	0.15–0.3 µM<br>12.5–25 µM<br>3.15–6.25 µM<br>0.8–1.6 µM<br>1.6–3.15 µM	*C. albicans*<br>*C. glabrata*<br>*C. tropicalis*<br>*C. neoformans*<br>*S. cerevisiae*	Silva et al., 2000;
12	Heliomicin (*Heliothis virescens*)	44(DKLIGSCVWGAVNYTSDCNGECKRRGYKGGHCGSFANVNCWCET)	2.5-5 µM<br>50 µM<br>0.1-0.2 µM<br>0.2-0.4 µM<br>0.4-0.8 µM<br>12 µM	*C. albicans*<br>*C. glabrata*<br>*N. crassa*<br>*F. culmorum*<br>*N. haematococca*<br>*C. neoformans*	Lamberty et al., 2001 and 2003; Mirza et al., 2018
13	Jelleine-I (*Apis mellifera*)	8(PFKLSLHL)	2.5-64 µM	*C. albicans*<br>*C. glabrata*<br>*C. tropicalis*<br>*C. krusei*<br>*C. parapsilosis*	Fontana et al., 2004; Jia et al., 2017 and 2018;
14	Jelleine-II (*Apis mellifera*)	9(TPFKLSLHL)	2.5 µM	*C. albicans*	Fontana et al., 2004
15	Lasioglossin III (*Lasioglossum laticeps*)	15 (VNWKKILGKIIKVVK)	10 µM	*C. albicans*	Chapuis et al., 2012

16	Lycosin-I (*Lycosa singoriensis*)	23(KGWFKAMKSIAKFIAKEKLKEHL)	8–128 μg/mL<br>16–256 μg/mL<br>32–256 μg/mL<br>128–512 μg/mL<br>128–512 μg/mL	*C. tropicalis*<br>*C. albicans*<br>*C. krusei*<br>*C. glabrata*<br>*C. parapsilosis*	Tan et al., 2018
17	MAF-1A (*Musca domestica*)	30(ESAPAPEVSGDAVFSAIQNGLKNLGNAFFW)	72.92 μg/mL	*C. albicans* ATCC 76615	Fu et al., 2009
18	Melectin (*Melecta albifrons*)	18(GFLSILKKVLPKVMAHMK)	8.3 μM	*C. albicans*	Slaninová et al., 2011; Liang et al., 2021
19	Metchnikowin (*Drosophila melanogaster*)	26(HRRQGPIFDTRPSPFNPNQPRPGPIY)	0.5-1.0 μM	*N. crussa*	Levashina et al., 1995
20	NDBP-5.7 (*Opisthacanthus cayaporum*)	13 (ILSAIWSGIKSLF)	25 μM	*Cryptococcus neoformans* & *Candida* sp.	Guilhelmelli et al., 2016; Nascimento et al., 2020
21	Oxysterlin 1 (*Oxysternon conspicillatum*)	39(GSKRWRKFEKRVKKIFEETKEALPVVQGVVAVATAVGRR)	50 μM	*Candida parapsilopsis* ATCC 22019	Toro Segovia et al., 2017
22	Oxysterlin 2 (*Oxysternon conspicillatum*)	55(GSKRWRKFEKKVKKALEDAKEKLQEERVQKIVEHTKEALPVIKAVATVVGVVGRR)	> 200 μM	*Candida parapsilopsis* ATCC 22019	Toro Segovia et al., 2017
23	Oxysterlin 3 (*Oxysternon conspicillatum*)	39(GSKRWRKFEKRVKKVFEHTKEALPVIQGVATVVGAVGRR)	> 200 μM	*Candida parapsilopsis* ATCC 22019	Toro Segovia et al., 2017
24	Oxysterlin 4 (*Oxysternon conspicillatum*)	62(GSKRWRKFEKKVKKALEDAKEKLQVSSSTIFFLFKLTFSGGIILLRKNEYRRSSNILKRLYR)	>200 μM	*Candida parapsilopsis* ATCC 22019	Toro Segovia et al., 2017
25	Papiliocin (*Papilio xuthus*)	38(RWKIFKKIEKVGRNVRDGIIKAGPAVAVVGQAATVVK)	5-20 μM	*A. flavus, A. fumigatus, A. parasiticus, C. albican, C. parapsilosis, M. furfur, T. beigelii,* & *T. rubrum*	Lee et al., 2010
26	Polybia-MPI (*Polybia paulista*)	14 (IDWKKLLDAAKQIL)	8 μM<br>16 μM	*C. albicans,*<br>*C. glabrata*	Wang et al., 2014
27	Polybia-MPII (*Pseudopolybia vespiceps testacea*)	14(IDWLKLGKMVMDVL)	EC_{50}: 12.9 μM<br>EC_{50}: 11.0 μM	*C. albicans*<br>*C. neoformans*	Silva et al., 2017
28	Profilin (*Spodoptera frugiperda*)	18(RYIYLSGTDRIIRAKLGK)	1-5 μM	*C. albicans,* & *C. tropicalis*	da Silva et al., 2020
29	Protonectin (*Agelaia pallipes*)	12(ILGTILGLLKGL)	8-128 μM	*C. albicans,*<br>*C. glabrata,*<br>*C. parapsilosis,*<br>*C. tropicalis,*<br>*C. krusei*	Wang et al., 2015; Qiu et al., 2017
30	Psacotheasin (*Psacothea hilaris*)	34(CIAKGNGCQPSGVQGNCCSGHCHKEPGWVAGYCK)	12.5 μM<br>6.25 μM<br>6.25 μM<br>12.5 μM	*C. albicans* ATCC 90028<br>*C. parapsilosis* ATCC 22019<br>*T. beigelli* KCTC 7707<br>*M. furfur* KCTC 7744	Hwang et al., 2010
31	Rondonin (*Acanthoscurria rondoniae*)	10 (IIIQYEGHKH)	8.37 μM<br><br><br>16.75 μM	*C. albicans* IOC 4558,<br>*C. glabrata* IOC 4565, &<br>*C. tropicalis* IOC 4560<br>*C. albicans* MDM8,<br>*C. guilliermondii* IOC 4557, & *C. parapsilosis* IOC 4564	Riciluca et al., 2012

32	Sarcotoxin Pd (*Paederus dermatitis*)	34(GWLKKIGKKIERVGQHTRGLGIAQIAANVAATAR)	18.62 µg/mL<br>25.26 µg/mL<br>22.3 µg/mL	*C. albicans*<br>*A. niger*<br>*A. fumigates*	Yazdi et al., 2013
33	Stomoxyn (*Stomoxys calcitrans*)	33(RGFRKHFNKLVKKVKHTISETAHVAKDTAVIAG)	0.78-1.56 µM<br>25-50 µM<br>100 µM	*C. neoformans*<br>*C. albicans* & *C. glabrata*<br>*S. cerevisiae*	Boulanger et al., 2002
34	Tenecin 1 (*Tenebrio molitor*)	43(VTCDILSVEAKGVKLNDAACAAHCLFRGRSGGYCNGKRVCVCR)	>100 µM	*C. albicans*	Lee et al., 1998
35	Termicin (*Pseudacanthotermes spiniger*)	36(ACNFQSCWATCQAQHSIYFRRAFCDRSQCKCVFVRG)	6-12 µM	*C. albican, C. glabrata*<br>*C. neoforman,* & *S. cerevisiae*	Lamberty et al., 2001
36	Thanatin (*Podisus maculiveris*)	21(GSKKPVPIIYCNRRTGKCQRM)	100 µM<br>5.0 µM	*N. crassa*<br>*F. oxysporum*	Fehlbaum et al., 1996; Mirza et al., 2018
37	ToAP2 (*Tityus obscurus*)	26 (FFGTLFKLGSKLIPGVMKLFSKKKER)	12.5 µM	*Cryptococcus neoformans* & *Candida* sp.	Guilhelmelli et al., 2016; do Nascimento et al., 2020
